# Growth inhibition of common neonatal pathogens differs between bovine lactoferrin products

**DOI:** 10.1099/jmm.0.002056

**Published:** 2025-08-29

**Authors:** Kyra P. Watral, David A. Kaufman, Timothy J. Boly, Jennifer R. Bermick

**Affiliations:** 1 Interdisciplinary Graduate Program in Immunology, University of Iowa, Iowa City, IA, USA; 2 Iowa Inflammation Program, University of Iowa, Iowa City, IA, USA; 3 Department of Pediatrics, Division of Neonatology, University of Virginia School of Medicine and Children’s Hospital, Charlottesville, VA, USA; 4 Department of Pediatrics, Division of Neonatology, University of Iowa, Iowa City, IA, USA

**Keywords:** antibacterial, bacteria, lactoferrin, neonate

## Abstract

**Introduction.** Infection is a leading cause of mortality during the first year of life, with the neonatal period being particularly high risk. It is critical to identify non-antibiotic approaches to improve neonatal infection outcomes due to the non-specific clinical signs of neonatal infection and negative consequences of early-life antibiotic exposure. Lactoferrin is a protein found in all mammalian milk that has a variety of antimicrobial properties. Clinical trials have shown that lactoferrin supplementation during the neonatal period decreases rates of sepsis.

**Knowledge Gap.** It remains unclear if there is an optimal lactoferrin preparation for human neonates.

**Aim.** Compare bacterial growth inhibition capabilities between different commercially available bovine-derived lactoferrin (bLF) preparations.

**Methodology.** This study uses a broth microdilution *in vitro* assay to directly compare the antibacterial effects and shelf stability of different bLF preparations against three common neonatal pathogens: *Escherichia coli*, *Klebsiella pneumoniae* and *Streptococcus pneumoniae*.

**Results.** Bacterial growth inhibition differed significantly between bLF manufacturers and between different bLF lots/batches from the same manufacturer. Approximately half of the bLF products demonstrated decreased bacterial growth inhibition capabilities within 7–14 days after solubilization.

**Conclusion.** These findings may help select optimal bLF products for clinical use in the neonatal population, but additional *in vivo* studies are needed to confirm our *in vitro* findings.

**Impact Statement**

This is the first study to directly compare the ability of different bovine lactoferrin products to inhibit the growth of common neonatal bacterial pathogens. Growth inhibition capabilities differed significantly between product manufacturers and lots/batches. Half of the products had decreased growth inhibition capabilities 7–14 days after solubilization. These findings could be important to consider when selecting a product for clinical implementation.

## Introduction

Infection is a leading cause of mortality during the first year of life [1]. The neonatal period, defined as the first 28 days after birth, is a particularly high-risk period for infection. Neonates experience a significantly higher infection burden than older children and adults, with preterm neonates experiencing the highest infection rates overall [2,5]. Neonatal infections often present with subtle and non-specific findings, resulting in delayed diagnoses and an overall mortality rate of 16% [6,7]. While infection prevention measures have made an impact in high-risk infants, further interventions are urgently needed. This is especially true for Gram-negative pathogens and for the youngest and smallest infants (≤1,000 g at birth), where the highest morbidity and mortality occur [8]. Additionally, clinicians often administer antibiotics to even mildly symptomatic neonates while awaiting culture results and prophylactically to neonates deemed to be at high risk of infection [9]. This is problematic, as early-life antibiotic exposure is associated with negative outcomes, including the selection of drug-resistant organisms [9], increased rates of late-onset sepsis [10], fungal infection [11], necrotizing enterocolitis (NEC) [11] and death [10] and a higher incidence of atopic disease later in life [12]. It is therefore critical to identify non-antibiotic approaches to improve neonatal infection outcomes.

Lactoferrin is an iron-binding protein found in all mammalian milk [13]. It has a variety of antimicrobial properties, which makes it an attractive infection prevention therapy for neonates [14,18]. Bovine-derived lactoferrin (bLF) has the strongest antibacterial activity of any species [19] and is the most common type of lactoferrin utilized in clinical trials [20,22]. bLF has antibacterial effects against a variety of neonatal sepsis-causing bacteria [23,25], and clinical trials have shown decreased rates of sepsis in preterm neonates supplemented with bLF [20,21]. However, it remains unclear if there is an optimal bLF preparation for human neonates, as antibacterial effects have never been directly compared between different bLF preparations. We therefore sought to determine if growth inhibition of common neonatal sepsis-causing bacteria differed between bLF products.

## Methods

### Bacterial culture

Lyophilized vials of bacterial strains were obtained from the American Type Culture Collection (ATCC). First-generation stocks were obtained according to the manufacturer’s instructions for each strain: *Escherichia coli* (ATCC 700973), *Klebsiella pneumoniae* (ATCC 13883) and *Streptococcus pneumoniae* (ATCC 49619). Stocks were reconstituted in 10% sterile glycerol (Sigma-Aldrich) and snap frozen in liquid nitrogen prior to long-term storage at −80 °C. For experimental use, a frozen glycerol stock of *E. coli* or *K. pneumoniae* was thawed in a 37 °C water bath, and 100 µl was used to inoculate 5 ml of tryptic soy broth (TSB, BD). These tubes were cultured overnight at 37 °C with shaking at 200 r.p.m., followed by subcultures at various dilutions in TSB (1 : 10, 1 : 20, 1 : 50 and 1 : 100) for 1 h at 37 °C with shaking at 200 r.p.m. *S. pneumoniae*-frozen stocks were thawed in a 37 °C water bath, and 100 µl was used to inoculate 5 ml of Todd–Hewitt broth (THB, BD) containing 0.1% yeast extract. These tubes were cultured overnight at 37 °C with 5% CO_2_ without shaking, followed by subcultures in THB plus 0.1% yeast extract (1 : 10, 1 : 20 and 1 : 50) for 1 h at 37 °C with 5% CO_2_ without shaking. OD600 measurements were obtained after subculture using a spectrophotometer (Molecular Devices). *E. coli* or *K. pneumoniae* subculture dilutions were streaked onto tryptic soy agar (TSA, BD) plates, and *S. pneumoniae* subculture dilutions were streaked onto TSA plates containing 5% defibrinated sheep blood (Thermo Scientific). Plates were incubated in a 5% CO_2_ incubator at 37 °C overnight to ensure no contamination was present and stocks remained a monoculture.

### Lactoferrin

Individual bLF preparations were gifted from each manufacturer and included Glanbia Nutritionals Bioferrin 1000 (Lot/Batch #1429493, Fitchburg, WI, USA), Glanbia Nutritionals Bioferrin 2000 (Lot/Batch #2889492, Lot/Batch #1940492001, Lot/Batch #2000491001, Fitchburg, WI, USA), Hilmar Apolactoferrin (Lot/Batch #PR1438, Lot/Batch #PR1439, Hilmar, CA, USA), Hilmar Lactoferrin 1000 (Lot/Batch #1258105, Lot/Batch #1189529, Lot/Batch #1188697, Lot/Batch #1258100, Hilmar, CA, USA), Tatua Lactoferrin (Lot/Batch #SP116101, Morrinsville, New Zealand), Vivinal Lactoferrin (Lot/Batch #103CBMC, Lot/Batch #105D5TZ, Friesland Campina, Amersfoort, The Netherlands) and Dicofarm BLF 100 (Lot/Batch #F0168, Rome, Italy). For experimental use, each preparation was dissolved in sterile TSB at an initial concentration of 400 mg ml^−1^. Vortexing at room temperature was enough to solubilize most preparations, but incubation at 37 °C at 200 r.p.m. for up to 30 min was required to fully solubilize some preparations. The final solutions were passed through a 0.2 µM filter to sterilize. The concentrated bLF stocks were stored at 4 °C until use. bLF stocks were used on the day of preparation, 7 days after preparation or 14 days after preparation to evaluate shelf stability.

### Broth microdilution

We utilized the broth microdilution for antibacterial testing recommended by the CLSI protocol [26] and described in Balouiri *et al*. [27] for the initial studies including *E. coli*, *K. pneumoniae* and *S. pneumoniae*. Serial twofold bLF dilutions were made to achieve concentrations ranging from 0.16 to 400 mg ml^−1^. One hundred fifty microlitres of each bLF dilution were plated in a 96-well flat bottom cell culture plate. Bacterial subcultures of *E. coli*, *K. pneumoniae* or *S. pneumoniae* at a McFarland Standard 0.5 (OD600 0.08–0.12) were diluted 1 : 150 (i.e. 33 µl bacterial stock in 4,967 µl broth), and 50 µl of the diluted bacteria was inoculated into separate wells containing the bLF dilutions. Wells containing sterile TSB without bLF were used as controls. Final volume in each well was 200 µl. Plates were incubated for 18 h at 37 °C with 5% CO_2_, and the OD600 was measured with a spectrophotometer as above as a surrogate for bacterial concentration. For these initial experiments, all conditions were performed in duplicate, and experiments were performed on two separate days. Subsequent experiments evaluated *E. coli* during the log-growth phase and were modified to include the addition of 50 µl of each bLF dilution and 50 µl of each diluted bacteria per well for a total volume of 100 µl. These plates were incubated at 37 °C with 5% CO_2_, and OD600 was measured hourly for 8 h with a spectrophotometer. All conditions were plated in triplicate, and experiments were performed on a single day.

### Statistical analysis

The inhibitory concentration 50 (IC50) was determined by plotting the log10(bLF concentration) on the x-axis and the OD600 on the y-axis in an XY table in GraphPad Prism (version 10.4.0). Nonlinear regression was performed using the following parameters: log(agonist) vs response, find ECanything, constraint *F*=50 and symmetrical (asymptotic) approximate 95% confidence intervals. Goodness of fit was determined by looking at the *R*^2^ and sum of square values. MIC was determined using the Gompertz model with symmetrical (asymptotic) approximate 95% confidence intervals [28]. A two-way ANOVA with Dunnett’s multiple comparison correction was used to compare bacterial concentrations over time between different groups. *P*-values<0.05 were considered significant.

## Results

A broth microdilution method was used to determine how different bLF preparations influenced overall bacterial growth by evaluating bacterial concentrations after completion of the log-growth phase (18 h). Three common neonatal pathogens were selected for these studies, including *E. coli*, *K. pneumoniae* and *S. pneumoniae. E. coli* containing the K1 capsular antigen is closely tied to neonatal sepsis and meningitis and has surpassed Group B *Streptococcus* as the leading cause of neonatal sepsis [29,31]. *K. pneumoniae* is the primary cause of neonatal sepsis in low- and middle-income countries [32], and *S. pneumoniae* results in severe clinical disease during neonatal sepsis [33,34]. All of the bLF products inhibited the growth of *E. coli*, *K. pneumoniae* and * S. pneumoniae*, although the IC50 and MIC values differed significantly between products (Table 1). *E. coli* was the most susceptible to bLF-induced growth inhibition with the lowest IC50 values across all products (Table 1). In contrast*, K. pneumoniae* and * S. pneumoniae* were more resistant to bLF-induced growth inhibition with higher IC50 values across all products (Table 1). Interestingly, different bLF brands were superior at inhibiting the growth of Gram-negative and Gram-positive bacterial strains, with Hilmar products demonstrating lower IC50 and MIC values for *E. coli* and *K. pneumoniae* and Vivinal products demonstrating lower IC50 and MIC values for *S. pneumoniae* (Table 1).

**Table 1.** Growth inhibition of common neonatal bacterial pathogens by different bovine lactoferrin products

Product	% Iron	*E. coli*		*K. pneumoniae*		*S. pneumoniae*	
		IC50	MIC	IC50	MIC	IC50	MIC
Glanbia Bioferrin 1000 (Lot/Batch #1429493)	0.01	1.5 (0.1–52)	61 (24–158)	129 (88–191)	926 (555–1,547)	201 (186–218)	254 (134–480)
Glanbia Bioferrin 2000 (Lot/Batch #2889492)	0.02	1.5 (0.1–55)	53 (22–127)	160 (122–211)	691 (469–1,019)	198 (180–218)	267 (177–403)
Tatua Lactoferrin (Lot/Batch #SP11610)	12	2.4 (0.3–19)	192 (31–1,186)	89 (57–138)	1,405 (747–2,642)	29 (22–39)	52 (30–87)
Vivinal Lactoferrin (Lot/Batch #103CBMC)	11	4.5 (0.9–23)	479 (81–2,849)	77 (35–244)	1,713 (615–4,772)	3.9 (3.4–4.4)	5.5 (3.6–8.4)
Vivinal Lactoferrin (Lot/Batch #105D5TZ)	11	6.7 (1.4–31)	1,098 (275–4,389)	76 (24–3,576)	1,950 (514–7,393)	4.1 (3.5–4.9)	5.9 (4.4–7.9)
Hilmar Apolactoferrin (Lot/Batch #PR1438)	1	2.8 (1.5–5.4)	32 (15–67)	33 (18–62)	891 (351–2,260)	12 (10–15)	23 (14–36)
Hilmar Lactoferrin 1000 (Lot/Batch #1258105)	9.9	1.3 (0.1–27)	471 (24–9,080)	32 (19–54)	923 (372–2,289)	28 (16–49)	29 (15–59)
Hilmar Lactoferrin 1000 (Lot/Batch #1258100)	12.7	3 (1.9–4.7)	51 (24–109)	19 (14–27)	305 (160–582)	179 (144–222)	305 (190–489)

IC50 in milligramme per millilitre with 95% confidence interval in parentheses; MIC in milligramme per millilitre with 95% confidence interval in parentheses; % iron, percentage of iron in product per manufacturer measurement.

**Fig. 1.**
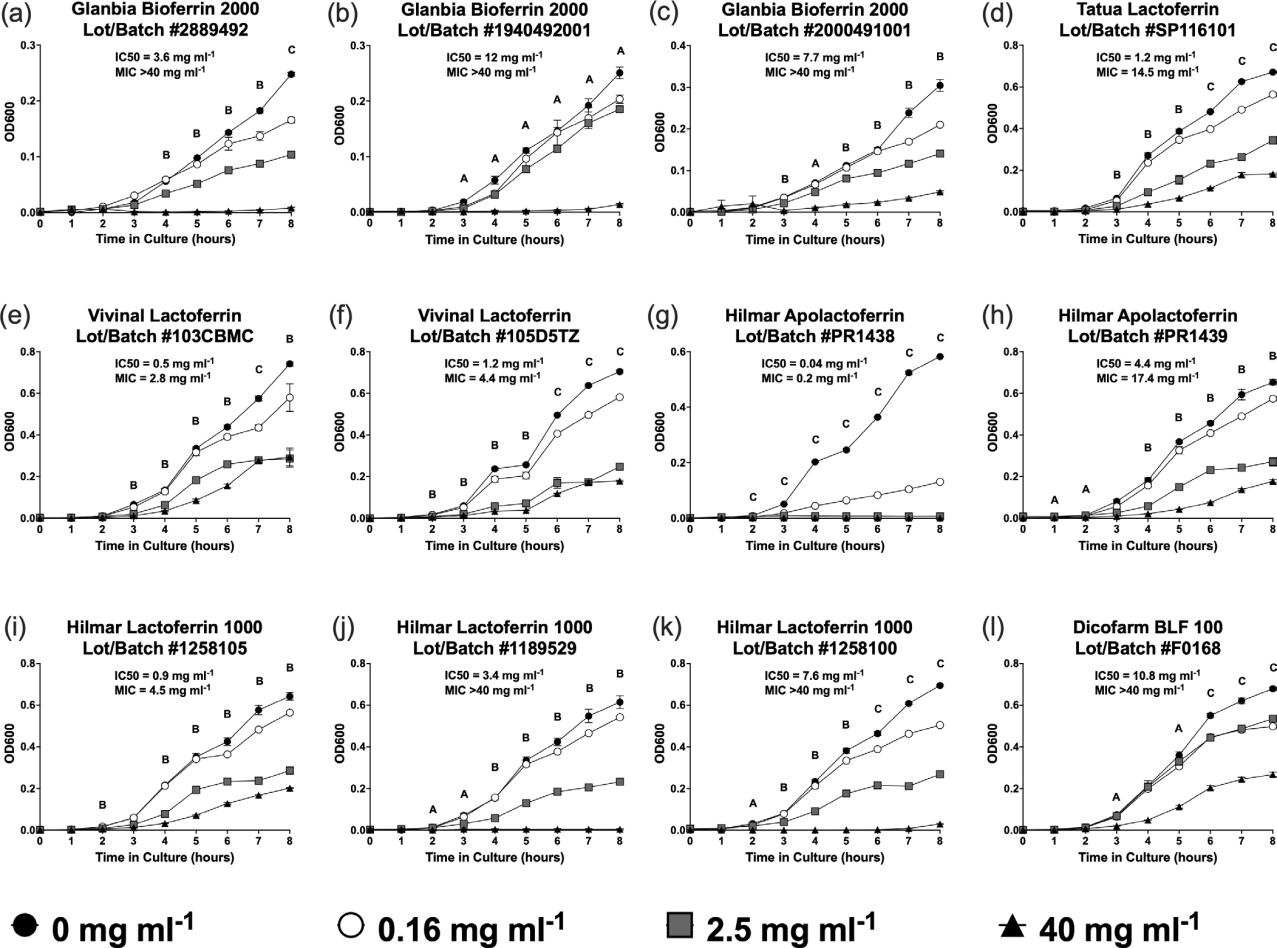
Bovine lactoferrin products demonstrate different dose-dependent *E. coli* growth inhibition *in vitro* during log phase. A 1 : 150 dilution of *E. coli* at McFarland Standard 0.5 was inoculated into twofold dilutions of different bovine lactoferrin products, and OD600 was measured hourly as a surrogate for bacterial concentration for 8 h. Bacterial growth is demonstrated for (a–c) Glanbia Nutritionals Bioferrin 2000 products, (**d**) Tatua Lactoferrin, (**e, f**) Vivinal Lactoferrin products, (**g, h**) Hilmar Apolactoferrin products, (**i–k**) Hilmar Lactoferrin 1000 products and (l) Dicofarm BLF 100. Nonlinear regression was used to determine IC50, and the Gompertz model was used to determine MIC at 8 h. Differences between bacterial OD600 readings were determined with two-way ANOVA with Dunnett’s multiple comparison correction. *n*=3 replicates per lactoferrin concentration and time point, all replicates plated on a single day. A=40 mg ml^−1^ with *P*<0.05 compared with 0 mg ml^−1^, B=40 and 2.5 mg ml^−1^ both with *P*<0.05 compared with 0 mg ml^−1^ and C=all concentrations with *P*<0.05 compared with 0 mg ml^−1^.

We next selected the most susceptible organism, *E. coli*, to evaluate the kinetics of bLF-induced bacterial growth inhibition during the log-growth phase. All product doses were compared with the positive control condition containing 0 mg ml^−1^ bLF at each time point, and whenever a dose of bLF was noted to be statistically significantly different from the positive control, all higher doses were also statistically significantly different (i.e. when 10 mg ml^−1^ had a *P*-value of <0.05 compared with 0 mg ml^−1^, doses of 20 and 40 mg ml^−1^ also had a *P*-value of <0.05 compared with 0 mg ml^−1^). All of the products demonstrated dose-dependent bacterial growth inhibition, although the degree of growth inhibition differed significantly between products (Figs 1a–l and S1A–L, Table S1, available in the online Supplementary Material). The time it took to reach maximal bacterial growth inhibition differed by product and dose, with higher doses achieving maximum growth inhibition at earlier timepoints for most of the products tested (Fig. S2A–L). Hilmar Apolactoferrin Lot/Batch #PR1438 demonstrated the most significant growth inhibition, with the lowest IC50 and MIC values (Figs 1g and S2G). Additional products contained IC50 and MIC values within the dosing range tested, including Tatua Lactoferrin Lot/Batch #SP11610, both Vivinal Lactoferrin products, Hilmar Apolactoferrin Lot/Batch #PR1439 and Hilmar Lactoferrin 1000 Lot/Batch #1258105 (Fig. 1d, e, f, h and i).

**Fig. 2.**
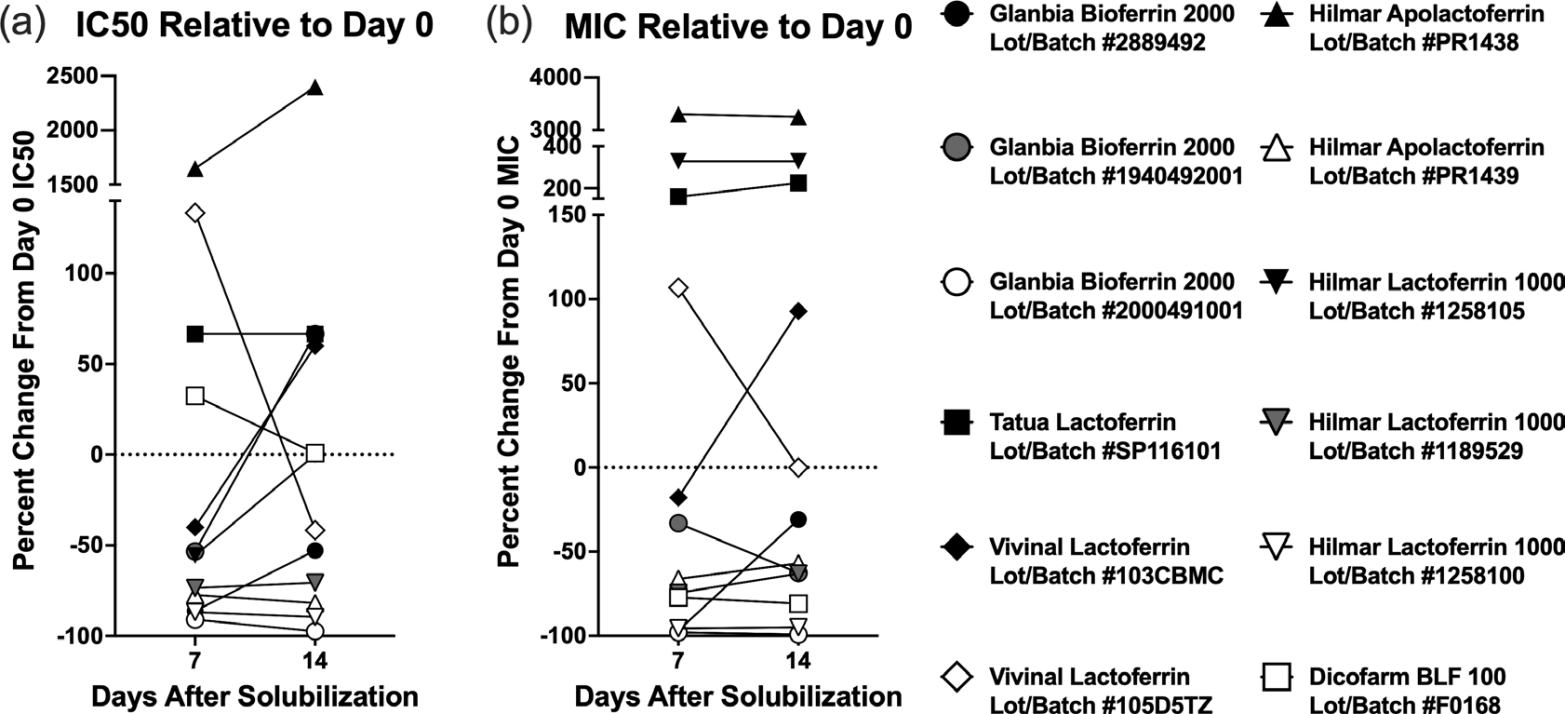
Most bovine lactoferrin products had declining bacterial growth inhibition 7–14 days after solubilization. A 1:150 dilution of *E. coli* at McFarland Standard 0.5 was inoculated into twofold dilutions of different bovine lactoferrin products at 0, 7 or 14 days after solubilization, and OD600 was measured after 8 h as a surrogate for bacterial concentration. (**a**) Nonlinear regression was used to determine IC50 for each product. IC50 on day 7 and day 14 was presented as per cent change from day 0 as [(day 7/14 IC50−day 0 IC50)/(day 0 IC50]×100. Positive values mean an increase in IC50 (signifies worse bacterial growth inhibition), while negative values mean a decrease in IC50 (signifies improved bacterial growth inhibition). The dotted line represents IC50 on day 0. (**b**) The Gompertz model was used to determine MIC for each product. MIC on days 7 and 14 was presented as per cent change from day 0 as [(day 7/14 MIC–day 0 MIC)/(day 0 MIC]×100. Positive values mean an increase in MIC (signifies worse bacterial growth inhibition), while negative values mean a decrease in MIC (signifies improved bacterial growth inhibition). The dotted line represents MIC on day 0. *n*=3 replicates per lactoferrin concentration and time point, all replicates plated on the same day.

We then wanted to investigate the shelf stability of each preparation to guide the implementation in a clinical setting. The same broth microdilution method was used to evaluate *E. coli* growth inhibition on the day of solubilization and 7 and 14 days after solubilization. Approximately half of the products demonstrated increasing IC50 and/or MIC values 7–14 days after initial solubilization, indicating a decline in bacterial growth inhibition capability (Fig. 2a, b). The products that showed decreasing bacterial growth inhibition capabilities over time included Tatua Lactoferrin Lot/Batch #SP116101, Vivinal Lactoferrin Lot/Batch #103CBMC, Vivinal Lactoferrin Lot/Batch #105D5TZ, Hilmar Apolactoferrin Lot/Batch #PR1438, Hilmar Lactoferrin 1000 Lot/Batch #1258105 and Dicofarm BLF 100 Lot/Batch #F0168 (Fig. 2a, b). Of note, all of the Glanbia products and some of the Hilmar products (Apolactoferrin Lot/Batch #PR1439, Lactoferrin 1000 Lot/Batch #1189529 and #1258100) had stable or improved bacterial growth inhibition capabilities up to 14 days after initial solubilization (Fig. 2a, b).

## Discussion

We demonstrated that the growth inhibition of common neonatal bacterial pathogens differed significantly between bLF products. These differences were noted both between product manufacturers and between lots/batches from the same manufacturer. bLF has well-described iron-dependent bacteriostatic mechanisms of bacterial growth inhibition [14,18]. Apolactoferrin is the low-iron conformation of lactoferrin (less than 20% iron saturation), with the greatest bacteriostatic activity [35]. It was therefore unsurprising that Hilmar Apolactoferrin Lot/Batch #PR1438 had some of the lowest IC50 and MIC values for the bacterial species tested. It was surprising that a different lot/batch of this same product (Hilmar Apolactoferrin Lot/Batch #1439) had significantly higher IC50 and MIC values compared with Lot/Batch #1438. We witnessed similar discrepancies between different lots/batches of Glanbia, Vivinal and Hilmar Lactoferrin products as well. While the total iron concentration was reported for each product, the lactoferrin iron saturation was not, so it is possible that products that demonstrated less bacterial growth inhibition had increased lactoferrin iron saturation even though the total iron concentration was not significantly different. Our experimental design did not account for the time between the initial date of product production and the time it was used experimentally, although all products were used prior to the manufacturer’s recommended expiration date. It is possible that products demonstrated a change in iron saturation levels over time, which could have contributed to the variability in bacterial growth inhibition between products. A recent study found that bLF retains equivalent functional properties during long-term storage as a powder for up to 9 years, somewhat mitigating this concern [36]. It is also important to consider the ubiquity of iron in our environment when considering the bacterial growth inhibition potential of bLF preparations. Environmental iron can be introduced into solutions through laboratory glassware, non-distilled liquids and metal equipment [37]. To minimize environmental iron contamination and increase the consistency of bLF’s bacteriostatic effects, manufacturers should consider the use of distilled liquids, single-use plastic consumables or acid-washed dedicated glassware in the manufacturing process. Another way to ensure consistency between bLF batches would be to test and report lactoferrin’s iron saturation for each batch rather than just the total iron concentration.

Another interesting observation was that bLF preparations from different manufacturers demonstrated superior growth inhibition of Gram-negative (Hilmar) and Gram-positive bacteria (Vivinal). bLF is known to bind to lipopolysaccharide (LPS) on the outer membrane of Gram-negative bacteria, which disrupts the integrity of the membrane and inhibits bacterial growth [18]. bLF exerts similar antibacterial activity against Gram-positive bacteria, binding to lipoteichoic acid (LTA) on the outer membrane to destabilize the membrane and inhibit growth [38]. While both LPS and LTA share similar physiochemical properties, they each have unique conformations that could favour enhanced binding by different compounds. Additionally, bLF has been shown to downregulate the *luxS* gene specifically in *S. pneumoniae*, which is necessary for biofilm formation [39]. It is therefore unclear if the superior *S. pneumoniae* growth inhibition demonstrated by Vivinal products will generalize to other Gram-positive bacteria or is specific for *S. pneumoniae*, possibly through the inhibition of the *luxS* gene. Future studies should test the ability of different bLF preparations to inhibit additional Gram-positive neonatal pathogens, including *Staphylococcus aureus*, *Staphylococcus epidermidis*, *Streptococcus agalactiae* and *Listeria monocytogenes*.

Approximately half of the bLF products demonstrated shelf stability up to 14 days after initial solubilization, with similar or lower bacterial IC50 and MIC values. The Glanbia products demonstrated the most consistent bacterial growth inhibition properties up to 14 days after solubilization, while the Vivinal, Tatua, Dicofarm and particular lots/batches of Hilmar products showed declining bacterial growth inhibition over time. We did not protect the solubilized products from light to mimic handling in a clinical setting, and while there is limited data about the photodegradation of lactoferrin compounds, other iron-associated complexes are known to be sensitive to photodegradation [40]. Storing the solubilized products in opaque containers may improve the shelf stability if some solutions are indeed sensitive to photodegradation, although additional studies are needed to understand the degree to which different bLF products are photosensitive as our study did not directly evaluate this.

Several clinical trials have supplemented preterm infants with bLF, with mixed results [41]. A meta-analysis found that bLF supplementation decreases rates of late-onset sepsis and NEC in preterm infants without adverse effects [41]. One of the largest clinical trials of bLF in preterm infants to date, the ELFIN trial, found that bLF supplementation did not reduce the risk of late-onset sepsis although multiple smaller clinical trials have shown a protective effect [41,42]. It should be noted that all of these clinical trials used different bLF manufacturers, doses and treatment regimens. Variability in bLF product used in each trial may explain the inconsistency in trial results and different reported outcomes. Despite these inconsistencies, there is strong evidence that bLF supplementation can decrease infection rates in preterm infants without negative side effects. However, the optimal bLF product for widespread clinical implementation remains unclear. When thinking about products for clinical implementation, it is important to consider efficacy, consistency and shelf stability. This study is the first to directly compare these factors between different bLF preparations. We believe that this is an important starting point when considering the best way to implement bLF supplementation into clinical practice.

## Conclusion

This was the first study to directly compare the ability of different bLF products to inhibit the growth of common neonatal bacterial pathogens. Bacterial growth inhibition differed significantly between bLF manufacturers and between different bLF lots/batches from the same manufacturer. About half of the bLF products demonstrated shelf stability up to 14 days after solubilization, although this varied by product. These findings are important to consider when selecting a bLF product for clinical implementation in the neonatal population, but additional *in vivo* studies are necessary to confirm our findings in a more complex model system.

## Supplementary material

10.1099/jmm.0.002056 Uncited Fig. S1.

10.1099/jmm.0.002056 Uncited Table S1.

## Abbreviations

ATCC: American Type Culture Collection
bLF: bovine-derived lactoferrin
IC50: inhibitory concentration 50
LPS: lipopolysaccharide
LTA: lipoteichoic acid
NEC: necrotizing enterocolitis
THB: Todd–Hewitt broth
TSA: tryptic soy agar
TSB: tryptic soy broth
