# Supplementary material for: Growth inhibition of common neonatal pathogens differs between bovine lactoferrin products
Source: J Med Microbiol. 2025 Aug 29;74(8):002056. doi: 10.1099/jmm.0.002056 (PMC12396925; doi:10.1099/jmm.0.002056)
Supplement: Uncited Fig. S1. [file jmm-74-02056-s001.pdf]

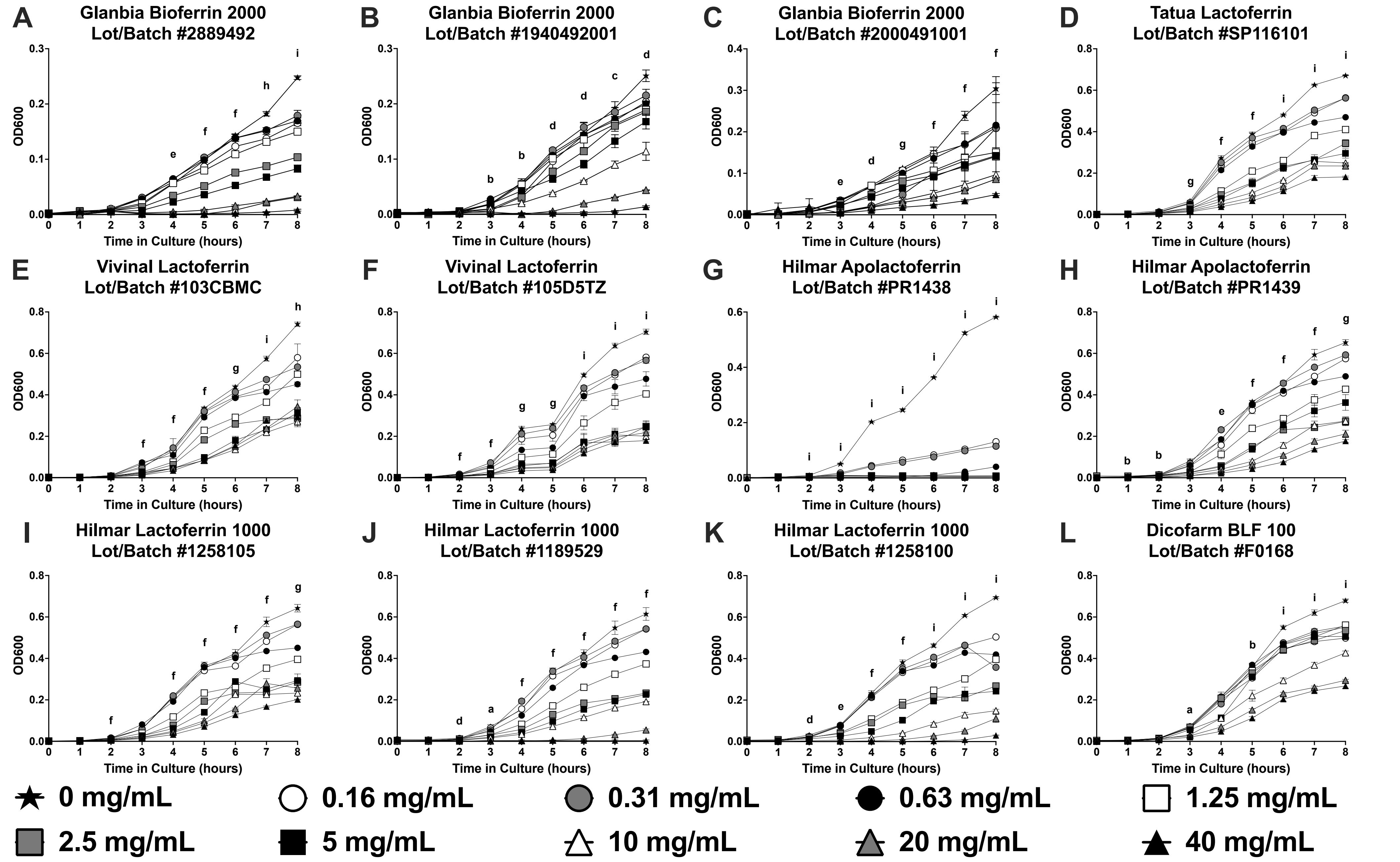

Supplementary Figure 1: Bovine lactoferrin products demonstrate different dose-dependent *Escherichia coli* growth inhibition *in vitro* during log-phase, all doses included. A 1:150 dilution of *Escherichia coli* at McFarland Standard 0.5 was inoculated into 2-fold dilutions of different bovine lactoferrin products and OD600 was measured hourly as a surrogate for bacterial concentration for eight hours. Bacterial growth is demonstrated for A-C) Glanbia Nutritionals Bioferrin 2000 products, D) Tatua Lactoferrin, E-F) Vivinal Lactoferrin products, G-H) Hilmar Apolactoferrin products, I-K) Hilmar Lactoferrin 1000 products and L) Dicofarm BLF 100. Differences between bacterial OD600 readings were determined with 2way ANOVA with Dunnett's multiple comparisons's correction. n=3 replicates per lactoferrin concentration and time point, all replicates plated on the same day. a = 40 mg/mL with $p<0.05$ compared to 0 mg/mL, b = concentrations 20 mg/mL and above (ie. 40 mg/mL) with $p<0.05$ compared to 0 mg/mL, c = concentrations 10 mg/mL and above (ie. 40 and 20 mg/mL) with $p<0.05$ compared to 0 mg/mL, d = concentrations 5 mg/mL and above (ie. 40, 20 and 10 mg/mL) with <0.05 compared to 0 mg/mL, e = concentrations 2.5 mg/mL and above (ie. 40, 20, 10 and 5 mg/mL) with $p<0.05$ compared to 0 mg/mL, f = concentrations 1.25 mg/mL and above (ie. 40, 20, 10, 5 and 2.5 mg/mL) with $p<0.05$ compared to 0 mg/mL, g = concentrations 0.63 mg/mL and above (ie. 40, 20, 10, 5, 2.5 and 1.25 mg/mL) with $p<0.05$ compared to 0 mg/mL, h = concentrations 0.31 mg/mL and above (ie. 40, 20, 10, 5, 2.5, 1.25 and 0.63 mg/mL) with $p<0.05$ compared to 0 mg/mL, i = all concentrations with $p<0.05$ compared to 0 mg/mL.

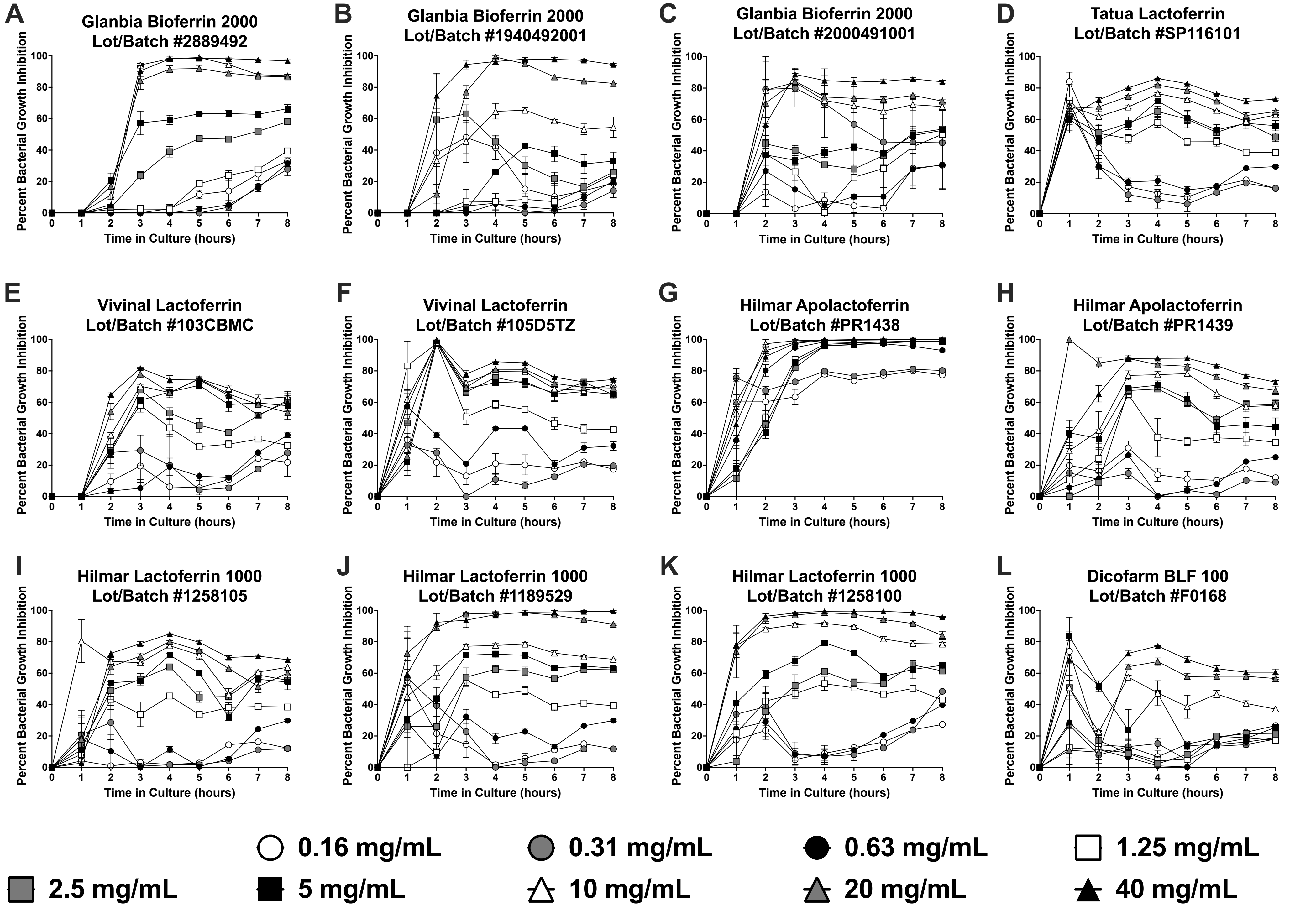

Supplementary Figure 2: Percent growth inhibition of *Escherichia coli* by bovine lactoferrin products *in vitro* during log-phase. A 1:150 dilution of *Escherichia coli* at McFarland Standard 0.5 was inoculated into 2-fold dilutions of different bovine lactoferrin products and OD600 was measured hourly as a surrogate for bacterial concentration for eight hours. Bacterial growth inhibition calculated as [(OD600 of 0 mg/mL positive control condition-OD600 of indicated bLF concentration)/(OD600 of 0 mg/mL positive control condition)] x 100. Percent bacterial growth inhibition is demonstrated for A-C) Glanbia Nutritionals Bioferrin 2000 products, D) Tatua Lactoferrin, E-F) Vivinal Lactoferrin products, G-H) Hilmar Apolactoferrin products, I-K) Hilmar Lactoferrin 1000 products and L) Dicofarm BLF 100. n=3 replicates per lactoferrin concentration and time point, all replicates plated on the same day.
